# Tracking People in a Mobile Robot From 2D LIDAR Scans Using Full Convolutional Neural Networks for Security in Cluttered Environments

**DOI:** 10.3389/fnbot.2018.00085

**Published:** 2019-01-08

**Authors:** Ángel Manuel Guerrero-Higueras, Claudia Álvarez-Aparicio, María Carmen Calvo Olivera, Francisco J. Rodríguez-Lera, Camino Fernández-Llamas, Francisco Martín Rico, Vicente Matellán

**Affiliations:** ^1^Grupo de Robótica, Universidad de León, León, Spain; ^2^Supercomputación Castilla y León (SCAYLE), León, Spain; ^3^Computer Science and Communications Research Unit, University of Luxembourg, Luxembourg City, Luxembourg; ^4^Robotics Lab, Universidad Rey Juan Carlos, Madrid, Spain

**Keywords:** convolutional networks, LIDAR, people tracking, robotics, cluttered environment

## Abstract

Tracking people has many applications, such as security or safe use of robots. Many onboard systems are based on Laser Imaging Detection and Ranging (LIDAR) sensors. Tracking peoples' legs using only information from a 2D LIDAR scanner in a mobile robot is a challenging problem because many legs can be present in an indoor environment, there are frequent occlusions and self-occlusions, many items in the environment such as table legs or columns could resemble legs as a result of the limited information provided by two-dimensional LIDAR usually mounted at knee height in mobile robots, etc. On the other hand, LIDAR sensors are affordable in terms of the acquisition price and processing requirements. In this article, we describe a tool named PeTra based on an off-line trained full Convolutional Neural Network capable of tracking pairs of legs in a cluttered environment. We describe the characteristics of the system proposed and evaluate its accuracy using a dataset from a public repository. Results show that PeTra provides better accuracy than Leg Detector (LD), the standard solution for Robot Operating System (ROS)-based robots.

## 1. Introduction

Detecting and tracking people are very useful capabilities for different systems, in particular for improving navigation in mobile robots and also to facilitate more socially acceptable robots, but also in security applications, for instance using biometric data (Ngo et al., 2015; Gavrilova et al., 2017) or safely using robotics platforms (Morante et al., 2015). There are many solutions in the literature that try to solve this problem using a multi-modal approach, typically with vision and range sensors (Arras et al., 2012), but these kinds of approaches are very expensive both from the point of view of the cost of the sensor and the computing capabilities needed for processing and integrating, and are more likely to generate contradictory information. For this reason, systems based only on range sensors are more desirable. Regarding the classifiers to process sensor data, Convolutional Neural Networks (CNNs) has emerged as a very popular solution (Long et al., 2015).

Laser Imaging Detection and Ranging (LIDAR) sensors are reliable and currently affordable range sensors that provide information about a dynamic environment at good rates (~ 20−30 Hz) that can be processed in real-time, as each scan consists of an array of just a few 100 integers.

Usually, mobile robots mount LIDAR scanners in a low position (~ 30 − 50 cm from the ground) to detect obstacles, which are used to build occupancy maps and to navigate. The information provided is an accurate estimate of the distance in precise angles (resolution of 0.5°). This means that objects such as tables or chair legs, trunks of plants, etc., may be easily confused with peoples' legs. It is also difficult to keep track of a particular person (pair of legs) in a crowded environment because many occlusions can happen.

Different solutions have been proposed previously in the literature to deal with the problem of tracking people using a 2D LIDAR scanner mounted on a mobile robot. Navigation in peopled, mapped, indoor environments has been recently reviewed (Rios-Martinez et al., 2015). The use of the geometric characteristics of human legs and the frequency and phase of walking motion have been tested (Lee et al., 2006), but cannot deal with partial occlusions, changes in peoples' speed, etc. In this article we are concerned with the specific problem of detecting pairs of legs (from a person) and being able to track them.

Early research (Schulz et al., 2003) applied Bayesian filtering to track different objects in the perceptual range of the robot to estimate the number of people in the current scan based on the number of moving local minima in the scan. Unfortunately, this supposes that people are continuously moving, giving poor results in cluttered environments (where the number of local minima is misleading).

Other research (Arras et al., 2012) proposed a solution based on AdaBoost to learn to detect individual legs. In a second level they proposed a multi-target tracking framework that uses leg observations to infer peoples' state of motion.

Another report (Leigh and Zhang, 2015) described a tracking method considering both legs, rather than individual ones. They proposed using a combination of Kalman filters to predict behaviors over consecutive scans and a Global Nearest Neighbor filter to solve the scan-to-scan data association problem. This solution has the drawback of using two different steps (prediction and association) that we are trying to solve with a one-step approach. Also, the authors acknowledge the problem of adapting their *ad-hoc* proposal. We propose that a more general system based on machine learning can be built.

Other researchers (Aguirre et al., 2014) have used Support Vector Machines to learn the different patterns of legs that can appear in robot surroundings corresponding to moving or still people. However, their approach is limited, tracking only a single person in a controlled scenario.

Other methods using several steps have been proposed. For instance, some have (Ondruska et al., 2016) deployed a Recurrent Neural Network (RNN) to filter an input stream of raw laser measurements in order to directly infer object locations, along with their identity in both visible and occluded areas. Others (Premebida et al., 2009) described a sensor fusion architecture. The fusion process occurs at the feature level, combining a LIDAR sensor and a camera for improving the detection system's reliability and accuracy. A different approach (Szarvas et al., 2006) presented a real-time pedestrian detection system utilizing a LIDAR-based object detector and CNN-based image classifier. The proposed method achieves a processing speed of over 10 frames/s speed by constraining the search space using the range information from the LIDAR.

Some solutions have also been developed for the Robot Operating System (ROS) framework (Quigley et al., 2009), the most popular framework for developing robotic applications. For instance, the *cob_people_perception* package allows to find leg-like patterns of laser scanner readings. This software is based on the LD approach and implementation. LD is the most popular package for tracking people by using a LIDAR sensor in ROS-based robots. It obtains incoming messages from the LIDAR scanner and uses trained data to classify the groups of laser records as possible legs. Detection is performed by a classifier using Random Trees implemented with the OpenCV API. However, LD has an important drawback; the project has not received continuous development and there is not a version of the software for the latest ROS versions.

Other ideas have been the use of heuristic knowledge, such as some research (Mashad Nemati et al., 2016) that proposes a technique that relies on the estimation of the reappearance event both in time and location. In the same way, a utility function to approximate and predict the trajectory of a walking partner has also been proposed (Morales et al., 2014).

In summary, we consider that learning techniques are a more general approach than heuristic or *ad-hoc* techniques for this problem. We also propose that a single shot approach can solve the problem in a more compact and consistent way than approaches based on several steps. Under this assumption, we propose a system based on CNNs developed by the Robotics Group at the University of León, named *PeTra*, for developing tracking systems based on LIDAR measurements. PeTra has been developed and tested on a mobile robot based on the ROS framework.

CNNs have been typically used on classification tasks, where the output to an image is a single class label (Lawrence et al., 1997; Krizhevsky et al., 2012; Simonyan and Zisserman, 2014). In our case, we want to label the pixels of an occupancy map (image), not the whole image. We have explored different alternatives and we have found that the ideas proposed in the U-Net architecture (Ronneberger et al., 2015) match with our requirements. The next section describes in depth the system built.

Evaluating neural networks requires a good validation dataset. Collecting and organizing a training set needs time and domain-specific knowledge. There is a large collection of robotic datasets available from various mobile robots, vehicles, or just handheld sensors, for instance the *Repository of robotics and computer vision datasets*^1^ for Mobile Robot Programming Toolkit (MRPT). However, most may not be suitable for training neural networks. For PeTra's validation, a dataset known as *Range-based people tracker classifiers Benchmark*^2^ (RRID:SCR_01574) was compiled.

In order to evaluate PeTra accuracy, we have compared its results with the ones obtained by LD, the most popular package to track people by using a LIDAR sensor in ROS-based robots. Evaluation was conducted in an indoor mock-up apartment located at the Robotics Lab at the University of León (Spain). A mobile robot called Orbi-One, with an on-board LIDAR sensor, was used to track peoples' locations using either PeTra or LD.

The rest of the paper is organized as follows. The next section describes the system that was built, including a detailed explanation of the architecture of the CNN used. Materials and methods used to evaluate the accuracy of PeTra are described in section 2. Section 3 shows the results of PeTra performance compared to LD. Section 4 discusses the above results. Finally, our contribution and the next steps foreseen are presented at section 5.

## 2. Materials and Methods

**Figure 1:**
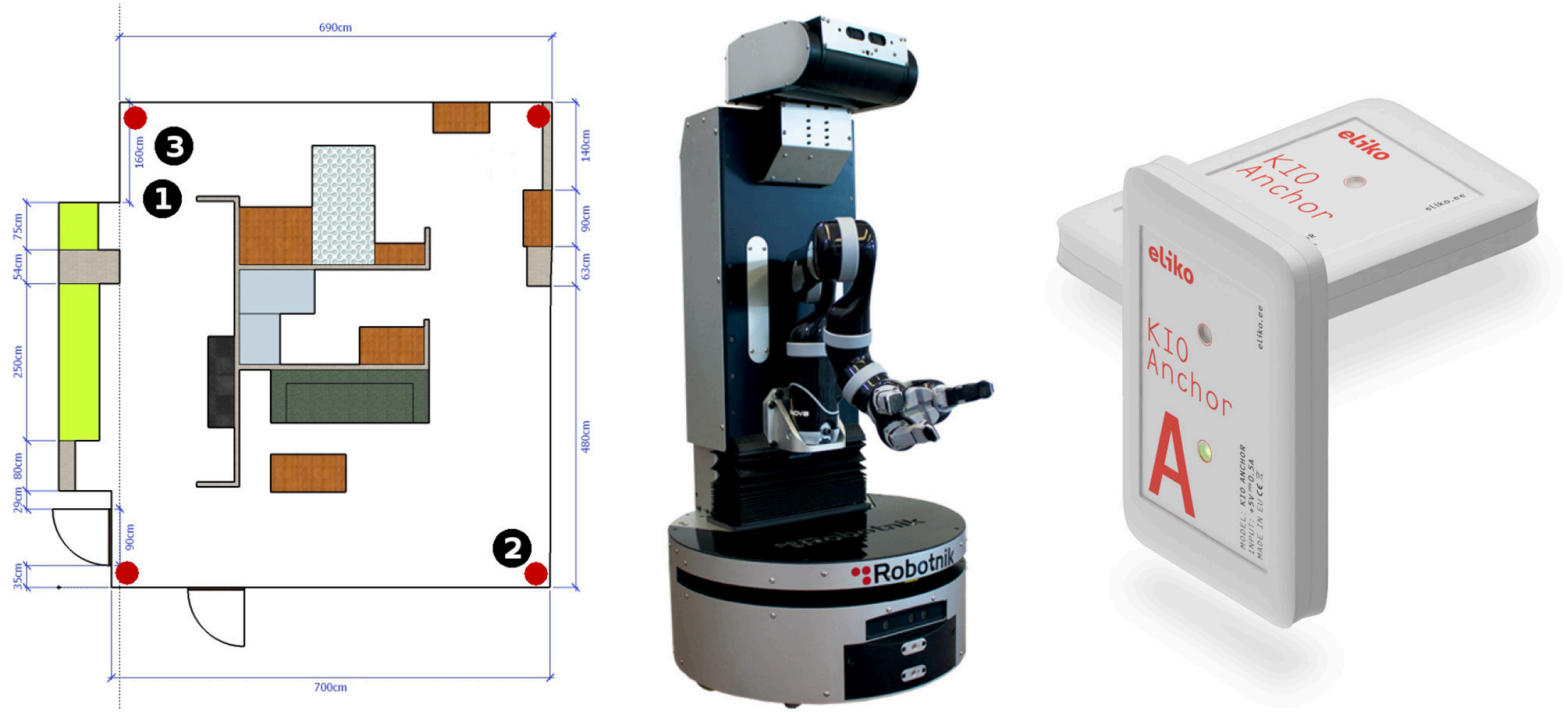
Robotics mobile lab plan **(left)**, Orbi-One robot **(center)**, and KIO RTLS anchors **(right)**.

A set of experiments was carried out to evaluate PeTra accuracy. They were conducted in the indoor mock-up apartment at Leon@Home Testbed^3^, a Certified Testbed^4^ of the European Robotics league (ERL) located in the Robotics Lab at the University of León. Its main purpose is to benchmark service robots in a realistic home environment. Figure 1, left shows the apartment plan.

Orbi-One robot, shown in Figure 1, middle, with an on-board LIDAR sensor, was used to track people either using PeTra or LD under different scenarios that will be described in section 2.5. Peoples' location was ascertained using a commercial Real Time Location System (RTLS) based on radio beacons shown in Figure 1, right. The location calculated by the RTLS was used as ground-truth data to calculate the error of PeTra and LD systems.

In the Youtube channel of the Robotics Group of the University of León, readers can find a video demonstration^5^ of PeTra and LD working as displayed on Rviz, a well-known tool for displaying robot information for the ROS framework.

Below, the main components of the experiment are described in depth as well as the methods used to evaluate accuracy.

### 2.1. Orbi-One Robot

Orbi-One is a service robot manufactured by Robotnik^6^. It accommodates sensors, such as a RGBD camera, a LIDAR sensor, and an inertial unit. It can operate a manipulator arm attached to its torso and it has a wheeled base for moving around the room. An Intel Core i7 CPU with 8 GB of RAM allows it to run the software to control the robot hardware. The software to control the robot hardware is based on a ROS framework.

### 2.2. KIO RTLS

A commercial RTLS (KIO) has been used to provide ground-truth data. KIO calculates the position of a mobile transceiver, called a *tag*, in a two- or three-dimensional space. In order to do so, KIO uses radio beacons, called *anchors*, that have been previously located in known positions in the surroundings. Red markers in Figure 1, left show the position of the 6 anchors used in the experiments described in the paper. They are attached at the ceiling of the mock-up apartment. The distribution of the anchors has been chosen following a previously established method (Guerrero-Higueras et al., 2017). Figure 1, right shows two KIO anchors.

The KIO tag was carried by the person to be tracked in our experiments. Location estimates provided by this system have an average error of ±30 cm according to the manufacturer's specifications. Calibrations done by the authors of this paper show that the error is higher in some areas and lower in others, but on average the claims of the manufacturer are correct (Guerrero-Higueras et al., 2017).

### 2.3. PeTra

PeTra is a tool for detecting and tracking people developed by the Robotics Group at the University of León. The system is based on a CNN which uses an occupancy map built from LIDAR measurements as input. We explored different alternatives and we have found that the configuration proposed in the U-Net architecture by Ronneberger et al. (2015) is the one that best matches with our requirements. U-Net architecture was developed for Biomedical Image Segmentation. It consists of a contracting path to capture context and a symmetric expanding path that enables precise localization that we need to look for an specific pattern (pair of legs) in the occupancy map.

#### 2.3.1. Neural Network Configuration

U-Net architecture was originally proposed to segment biomedical images. We have adapted it to LIDAR map “images.” Basically, the architecture is an evolution of the full CNN proposed in Long et al. (2015). It consists of supplementing the usual contracting network by successive layers, where pooling operators are replaced by upsampling operators. Hence, these layers increase the resolution of the output. In this way, the contracting path captures context and a symmetric expanding path enables precise localization of targets.

**Figure 2:**
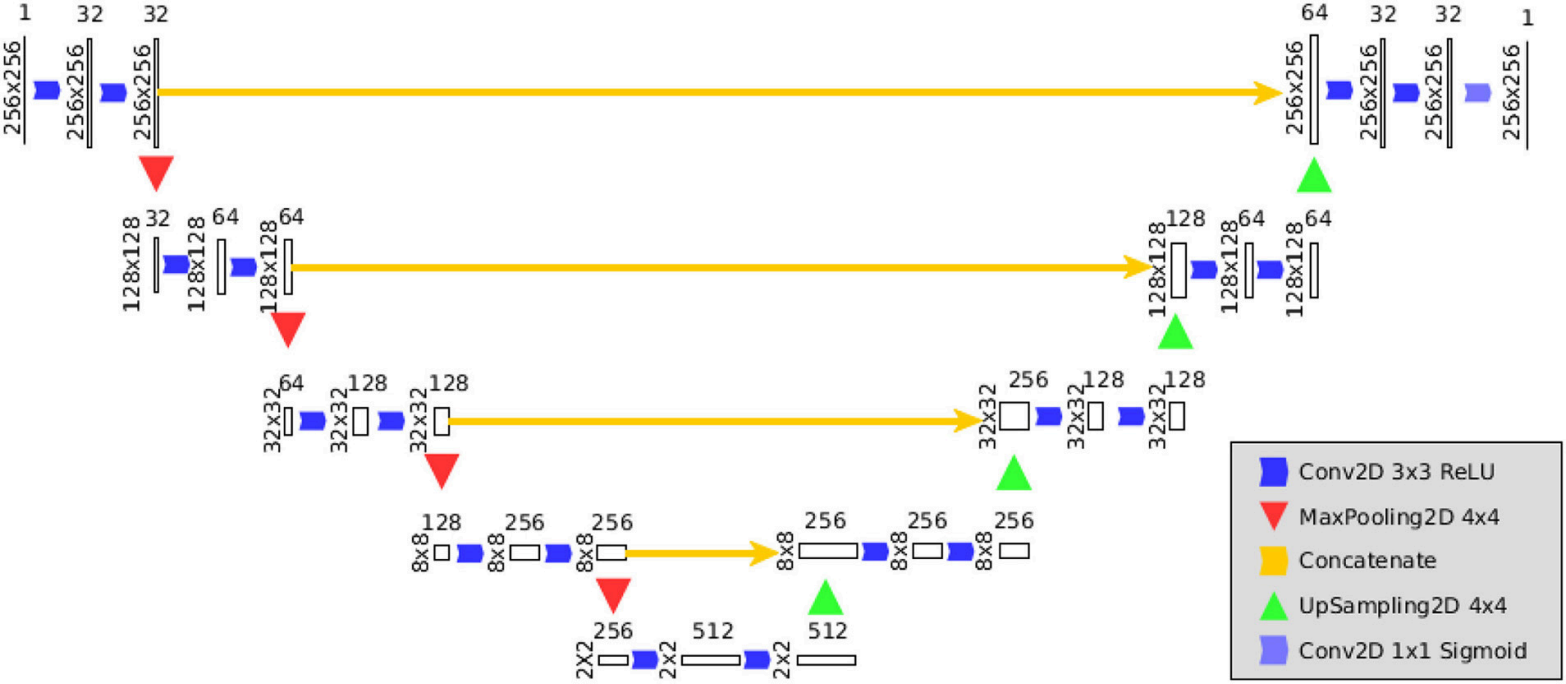
Architecture of the CNN used by PeTra.

Figure 2 illustrates the architecture of the CNN embedded in PeTra. It is inspired by the design in Ronneberger et al. (2015), adapting sampling sizes. Basically, the PeTra network consists of a contracting path on the left side and an expansive path on the right side of the picture. The contracting path consists of the repeated application of two 3 × 3 convolutions, followed by a Rectified Linear Unit (ReLU) and a 4 × 4 max pooling operation with stride 2 for downsampling. At each downsampling step we doubled the number of feature channels. Every step in the expansive path consists of a 4 × 4 up-convolution that reduces the number of feature channels, a concatenation with the corresponding feature map from the contracting path, and two 3 × 3 convolutions, each followed by a ReLU. At the final layer a 1 × 1 convolution is used to map each 64-component feature vector to the desired number of classes.

In order to define the input of the network, we have projected the range measurements into a two dimensional map centered around the robot. This occupancy map is defined as a 256 × 256 matrix, with a resolution of about 2 cm. The occupancy map presented in this work is not a standard occupancy map containing probability values as known in the literature. The values of each cell could be:

**0:** meaning either the LIDAR scan went through it without detecting any obstacle, or it did not go through that position during the reading due to occlusions or being out of range (LIDAR range is 240 degrees in the front of the robot),
**1:**, meaning an obstacle was found in that position.

#### 2.3.2. Operation

**Figure 3:**
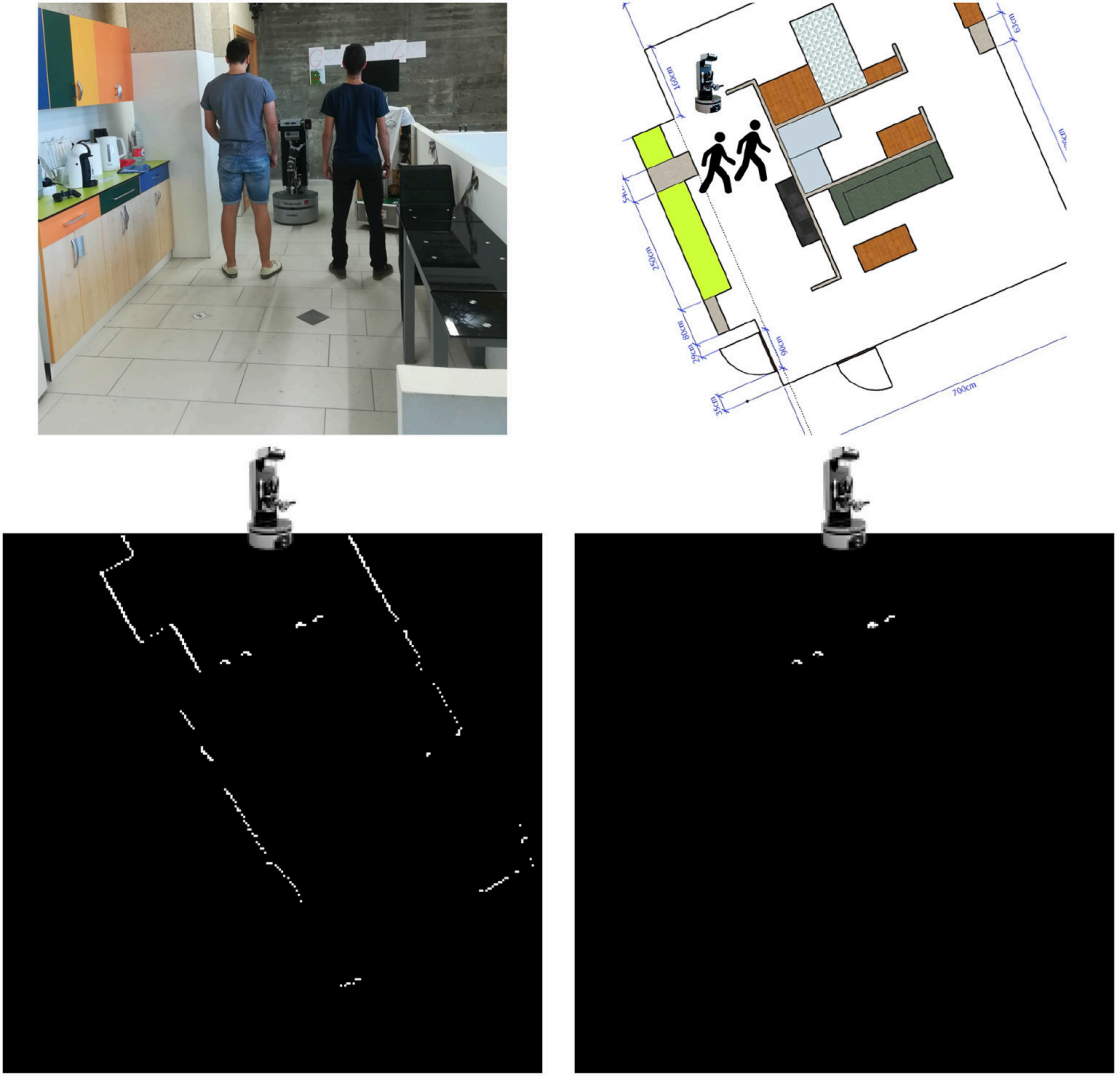
**Top left:** Orbi-One and two people at the kitchen of the mock-up apartment. **Top right:** same situation over the apartment plan. **Bottom left:** occupancy map built from LIDAR's readings on the above situation. **Bottom right:** PeTra's output after processing the above occupancy map.

**Figure 4:**
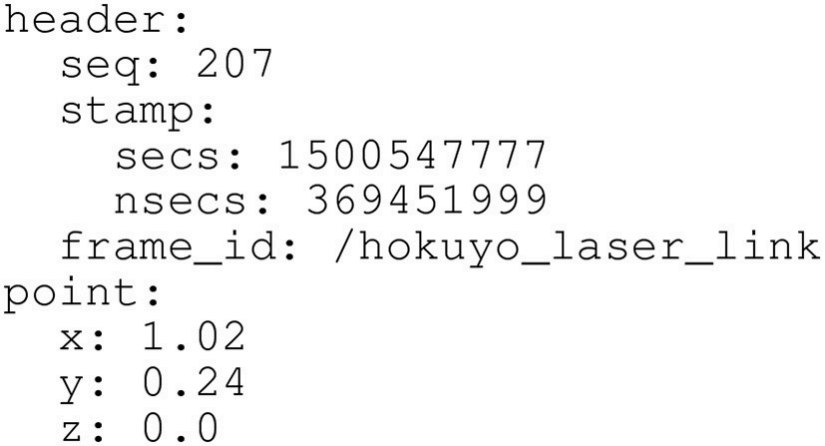
Location message (PointStamped message, in ROS terminology) published at the /person topic. It includes a header with a nanosecond-precise timestamp and some identification data as well as a body with the Cartesian coordinates of the location.

PeTra has been developed and tested on a mobile robot based on the ROS framework. It has been designed as a ROS node with the CNN described at section 2.3.1 embedded on it. Once the network is trained, the system is capable of performing the following steps in real time:

1. First, the data provided by the LIDAR sensor is processed to build a two-dimensional occupancy map in front of the robot. Figure 3, top left shows Orbi-One and two people at the study area. Figure 3, top right shows the same situation over the apartment plan. Figure 3, bottom left illustrates the occupancy map obtained from the LIDAR's readings on the above situation. The occupancy map is presented as a 256 × 256 picture, where white pixels denote positions where the LIDAR scan found an obstacle and black pixels denote positions where either the LIDAR scan went through without detecting any obstacle or did not go through that position.
2. Then, the occupancy map of the previous step is given to the network as input data. The network produces a second occupancy map representing the zones where legs have been detected. Figure 3, bottom right shows the network output after processing the occupancy map shown in Figure 3, bottom left.
3. Finally, a mass center calculation returns peoples' locations, which is published as a location message in a specific topic. Location messages include, among other data, the location coordinates and a timestamp that is precise to the nanosecond, as shown in Figure 4.

**Figure 5:**
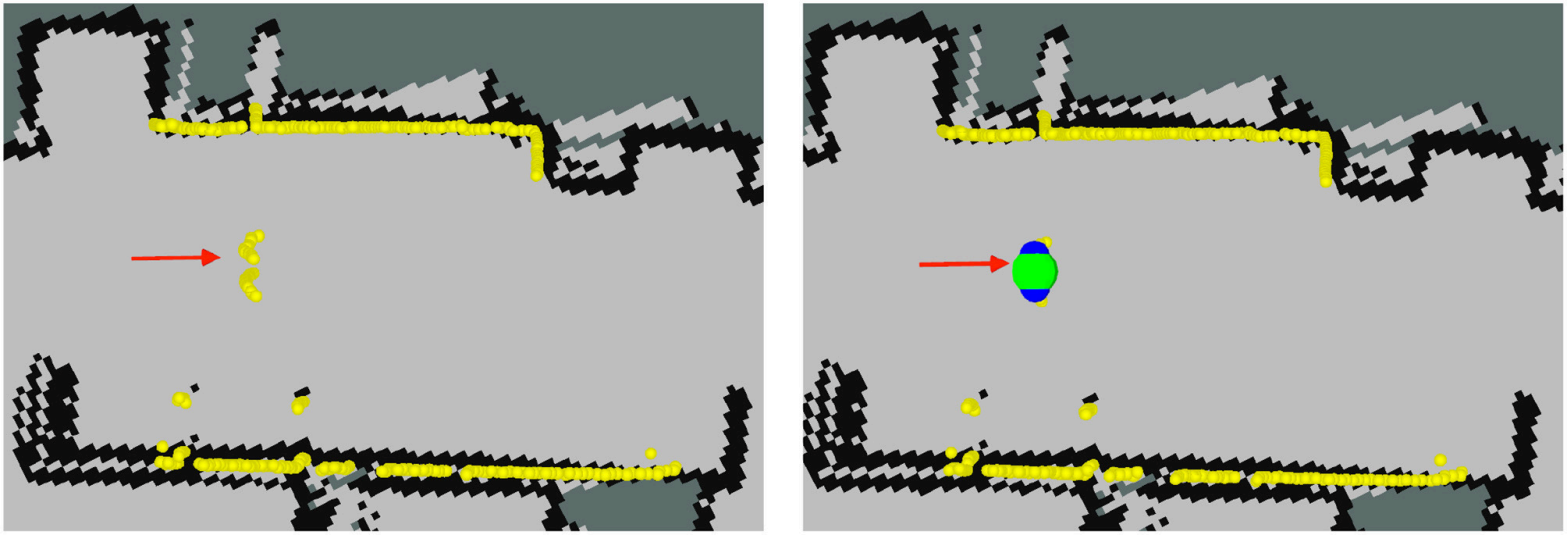
Information of the robot as displayed on Rviz (running PeTra in the right and without running it to the left): Yellow markers show LIDAR readings; the red arrow shows Orbi-One's location and orientation, the beginning of the arrow matches the robot location; blue markers show leg location estimates; and the green marker shows the person center.

Location messages published by PeTra will be used to evaluate its performance by comparing it with ground-truth data. PeTra also publishes visualization Marker messages to indicate where detection happened. These markers can be displayed on Rviz, as shown on Figure 5.

#### 2.3.3. Neural Network Training

A CNN embedded in PeTra was trained using data gathered at the Robotics Lab of the University of León (see Figure 1, left). To get training data a single person walked in a straight line toward the robot and then turned around to move away while the robot remained still. This situation was repeated in two locations at the mock-up apartment, the kitchen and the living room.

**Figure 6:**
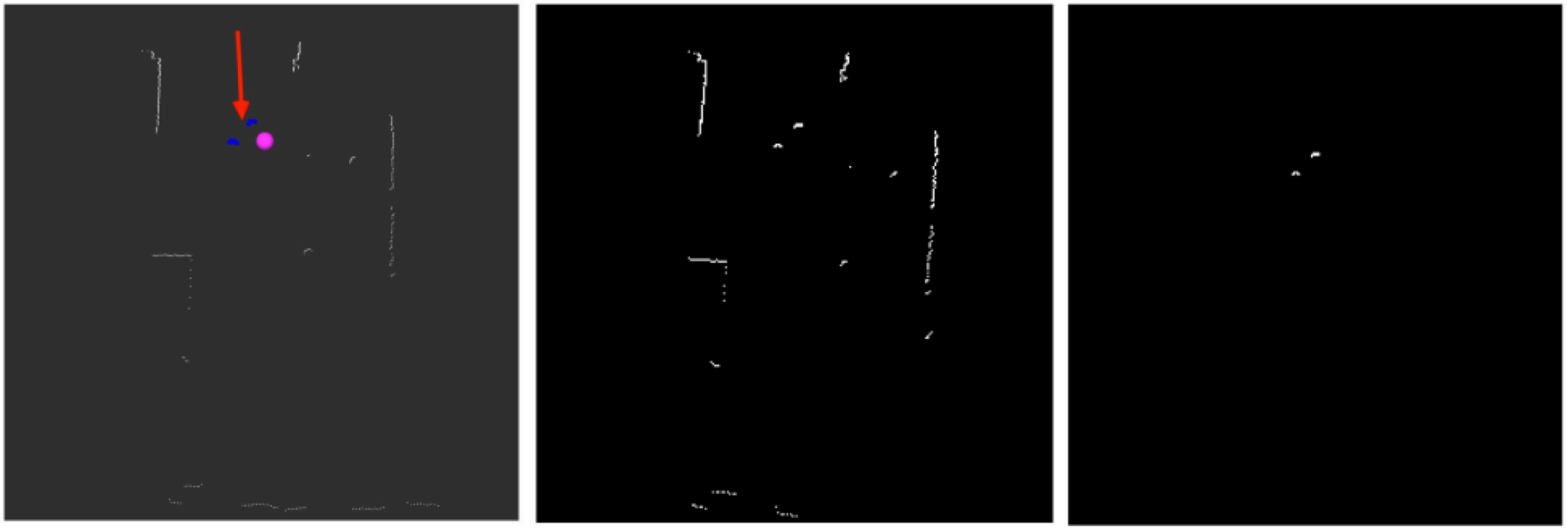
Information of the robot as displayed on Rivz **(left)**: white points show LIDAR readings; the red arrow shows Orbi-One's location and orientation, the beginning of the arrow matches the robot location; the pink marker shows the KIO location estimate; and blue markers show the LIDAR readings close to the PeTra location estimate. Occupancy map built from LIDAR readings **(middle)**. Labeled data **(right)**.

To label training data, KIO location estimates were used. In order to do so, for each LIDAR scan, an occupancy map was created. Then, discarding all LIDAR readings close to the KIO location estimate, leg position were labeled. To illustrate this process Figure 6 shows the labeling from a specific LIDAR scan. Figure 6, left shows some information of the robot as displayed on Rivz. LIDAR's readings are shown as white points. The red arrow shows Orbi-One robot's pose (location and orientation). The robot location matches the beginning of the arrow. The pink marker shows the KIO location estimate of the person in the scene. Blue markers show the LIDAR readings close to the PeTra location estimate. Figure 6, middle shows the occupancy map created from the above data. Figure 6, right shows the labeled data by gathering the LIDAR readings close to the KIO location estimate.

The training dataset includes 2,790 pairs of images (occupancy- and labeled-map). Two thousand two hundred and twenty four were used to train the network and 550 to test it. PeTra training data is available at the University of León Robotics group website^7^ As mentioned in the introduction, for PeTra evaluation, in order to ensure an optimal generalization, a different dataset was used including situations at different environments with several people in the scene, as will be explained later.

### 2.4. Leg Detector (LD)

LD is a standard ROS package which takes messages published by a LIDAR sensor as input and uses a machine learning-trained classifier to detect groups of LIDAR readings as possible legs. The code is available in a public repository^8^, but is unsupported at this time.

**Figure 7:**
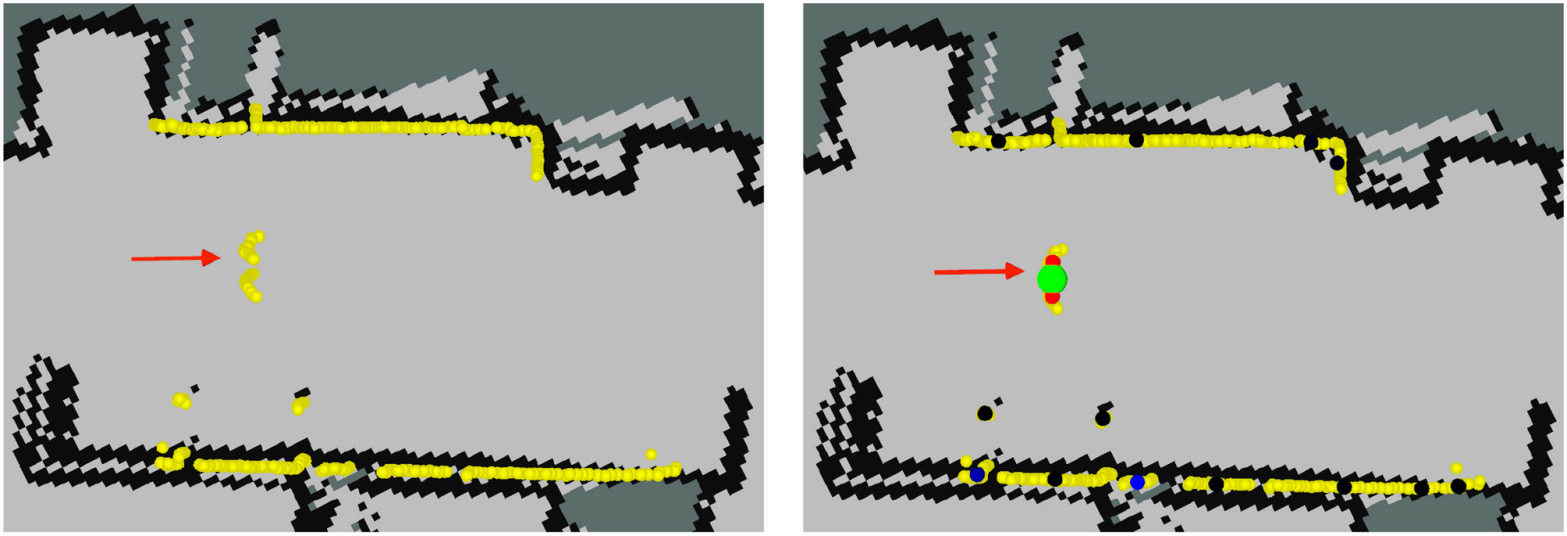
Information of the robot as displayed on Rviz (running LD in the right and without running it to the left): Yellow markers show LIDAR readings; the red arrow shows Orbi-One's location and orientation, the beginning of the arrow matches the robot location; black-to-blue markers show leg location estimate, close-to-black markers represent a small chance that it is a real leg, close-to-blue markers represent a high probability; red markers show paired legs; and the green marker shows the person center.

Figure 7 shows LD location estimates as displayed on Rviz. It publishes the location for the individual legs. Markers colored in a black-to-blue gradient show leg location estimates. A close-to-black marker represents a small chance that it is a real leg while a close-to-blue marker represents a high probability. Some false positives appear in Figure 7, right, see the blue marker close to the wall. LD can also attempt to pair the legs together (displayed as red markers on Rviz) and publish their average as an estimate of where the center of the person is displayed on Rviz as a green marker.

### 2.5. Evaluation

A complete dataset, different than the training dataset, has been used for PeTra evaluation. This section describes how the evaluation dataset has been built. Later, the evaluation method is proposed.

#### 2.5.1. Data Collection

**Figure 8:**
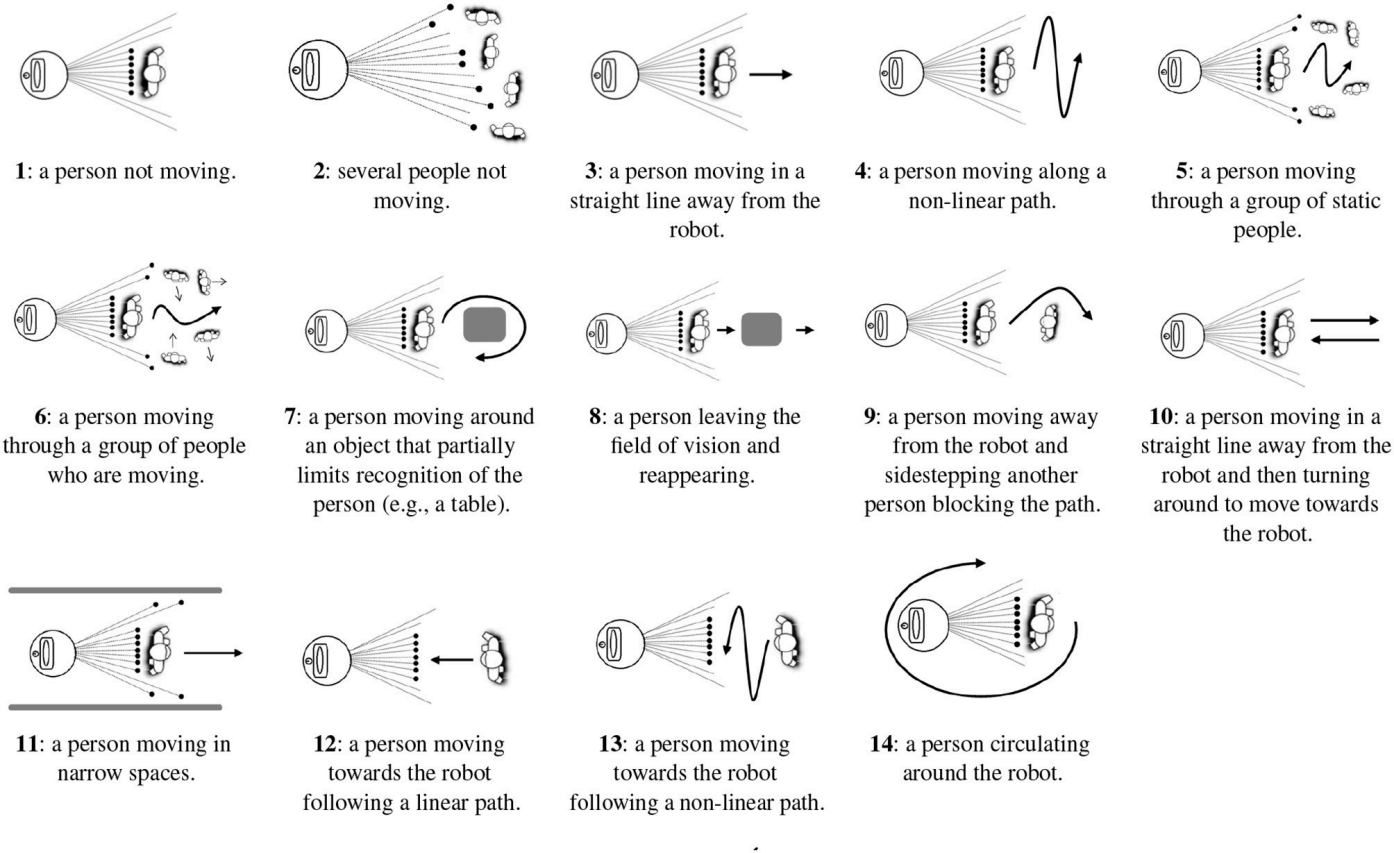
Recognition situations recorded as shown at Álvarez-Aparicio et al. (2018).

Evaluation data, different than training data to ensure generalization, is needed to evaluate PeTra. Therefore, *Range-based people tracker classifiers Benchmark* dataset^2^ (RRID:SCR_01574) was used. The dataset has different versions and data from version 2 were used in the evaluation. Data were gathered in 14 different situations, where the robot stood still as one person, carrying a KIO tag, moved around it. Situations represent different human-robot interactions that may occur in robotics competitions such as ERL^9^ or RoboCup^10^, in particular in the “Following and Guiding” test where a robot has to follow an operator in a cluttered environment. Figure 8 illustrates each of these situations. Three different locations were defined (black numbered markers in Figure 1, left show Orbi-One locations during the data gathering) resulting in 42 scenarios (14 situations × 3 locations).

Recently, a new version of the dataset was released (version 3). These data were also used in the evaluation. New data were gathered according to situations 5 and 10 (see Figure 8) on three different locations (kitchen, living room and bedroom), but in this case Orbi-One was not still but was instead moving around the room.

Rosbag files were used to record the state of the robot by gathering data from the robot's sensors and actuators such as LIDAR scans, odometry data, etc. A rosbag file is equivalent to a recording of the state of the robot in a period. A rosbag file was created for each scenario. In all of them, the person to be tracked was carrying a KIO tag in order to get his real location. Each rosbag file contains the following data:

– LIDAR sensor data which include, among other information, the following: acquisition time of the first ray for each scan, start/end angle, angular distance between measurements, and range data.
– PeTra location estimates which include a position [x, y, z] and a timestamp.
– Location estimates calculated by LD including data for individual legs and paired legs which will be compared to ground-truth data.
– Locations provided by KIO RTLS which also include a position and a timestamp.
– Useful information such as map information, odometry of the robot base, and transformation information, respectively.

Further information about the dataset may be found at Álvarez-Aparicio et al. (2018).

#### 2.5.2. Evaluation Method

In order to evaluate PeTra accuracy, a comparison with LD was done. In order to empirically decide which one offers the best results, location estimates of people from both systems can be compared to the ground-truth data provided by KIO RTLS. The accuracy error of both PeTra (*e*_*PeTra*_) and LD (*e*_*LD*_) in a specific instant of time has been calculated as the euclidean distance between their location estimates (*l*_*PeTra*_ and *l*_*LD*_) and ground-truth data provided by KIO-RTLS (*l*_*KIO*_). A comparison may be done just in case there is a valid location estimate from PeTra or LD, otherwise *e*_*PeTra*_ and *e*_*LD*_ will respectively get a maximum value. Equations 1 and 2 show *e*_*PeTra*_ and *e*_*LD*_ calculation,

(1) $$e_{PeTra}\;=\{\begin{array}{lll} \text{Max}.\text{ range value} & \text{if}∄\;l_{PeTra} & \;\\ d(l_{PeTra},l_{KIO})=\sqrt{\sum_{i=1}^{n}{\left(l_{PeTra_{i}}-l_{KIO_{i}}\right)}^{2}} & \text{if}\exists \;l_{PeTra} & \; \end{array}$$

(2) $$e_{LD}=\{\begin{array}{ll} \text{Max}.\text{ range value} & \text{if}∄\;l_{LD}\\ d(l_{LD},l_{KIO})=\sqrt{\sum_{i=1}^{n}{\left(l_{LD_{i}}-l_{KIO_{i}}\right)}^{2}} & \text{if}\exists l_{LD} \end{array}$$

where *n* is the number of dimensions considered. In our experiments, only *X* and *Y* coordinates are considered. The *Z* coordinate is constant since a mobile robot moves on the ground. Ground-truth data location estimates (*l*_*KIO*_) are provided as Cartesian coordinates by KIO RTLS. PeTra and LD location estimates (*l*_*PeTra*_ and *l*_*LD*_) are also provided as Cartesian coordinates but using the LIDAR location as the coordinates' origin.

The evolution of *e*_*PeTra*_ and *e*_*LD*_ over time was used to decide the system which works better. The accuracy error was calculated for the period of time covered for each Rosbag file included in the dataset.

Regarding the above, there are two important issues to deal with as detailed in Álvarez-Aparicio et al. (2018). First, KIO, PeTra and LD use their own coordinate origins to represent locations. In order to compare these locations they ought to be represented using the same coordinate origins. The *tf* package, see Foote (2013), included in the ROS framework core, is in charge of transforming KIO, PeTra and LD location estimates, allowing for easy comparison. *tf* operates with a central server that contains all transform information. On the other hand, each message published for the recorded topics has its own timestamp with precision to the nanosecond, so comparing locations in a concrete instant of time may not be an easy task. A synchronization method is needed to compare measurements from different topics. To synchronize data in our experiments, we have used the timestamp of the PeTra location estimates and then we have selected the closest measure in time from both LD and KIO.

## 3. Results

The accuracy of PeTra has been evaluated vs. LD accuracy applying the described method. Table 1 shows the mean error and standard deviation for the 14 situations of the second version of the dataset. As can be seen in the table, the average error of *e*_*PeTra*_ at locations 1, 2, and 3, are 0.17, 0.43, and 0.20 m respectively; it is lower than *e*_*LD*_ at all locations (0.30, 0.75, and 0.49 m respectively).

**Table 1:** Mean error and standard deviation (m) using data from version 2 of the dataset.

	**Location 1**		**Location 2**		**Location 3**	
**Situation**	${\bar{X}}_{e_{PeTra}}$	${\bar{X}}_{e_{LD}}$	${\bar{X}}_{e_{PeTra}}$	${\bar{X}}_{e_{LD}}$	${\bar{X}}_{e_{PeTra}}$	${\bar{X}}_{e_{LD}}$
1	0.02 (±0.02)	0.21 (±0.04)	1.40 (±0.03)	1.16 (±0.03)	0.03 (±0.01)	0.52 (±0.09)
2	0.48 (±0.47)	0.17 (±0.11)	1.25 (±0.29)	1.51 (±0.19)	0.48 (±0.35)	0.33 (±0.08)
3	0.08 (±0.10)	0.32 (±0.15)	0.06 (±0.03)	0.62 (±0.40)	0.06 (±0.02)	0.20 (±0.08)
4	0.09 (±0.06)	0.30 (±0.15)	0.36 (±0.50)	0.85 (±0.39)	0.15 (±0.12)	0.32 (±0.11)
5	0.43 (±0.12)	0.35 (±0.13)	0.95 (±0.46)	0.84 (±0.30)	0.38 (±0.07)	0.54 (±0.10)
6	0.16 (±0.22)	0.21 (±0.10)	0.55 (±0.44)	0.63 (±0.44)	0.36 (±0.29)	0.61 (±0.27)
7	0.18 (±0.22)	0.39 (±0.25)	0.20 (±0.14)	0.95 (±0.52)	0.21 (±0.13)	0.24 (±0.12)
8	0.11 (±0.14)	0.26 (±0.11)	0.08 (±0.07)	0.73 (±0.28)	0.08 (±0.06)	0.39 (±0.16)
9	0.29 (±0.18)	0.25 (±0.15)	0.69 (±0.60)	0.56 (±0.42)	0.41 (±0.20)	1.78 (±2.89)
10	0.10 (±0.06)	0.36 (±0.16)	0.10 (±0.07)	0.71 (±0.24)	0.19 (±0.13)	0.50 (±0.16)
11	0.05 (±0.03)	0.26 (±0.08)	0.08 (±0.18)	0.18 (±0.06)	0.08 (±0.09)	0.28 (±0.05)
12	0.04 (±0.02)	0.27 (±0.16)	0.06 (±0.05)	0.53 (±0.27)	0.04 (±0.04)	0.55 (±0.13)
13	0.12 (±0.11)	0.49 (±0.28)	0.13 (±0.10)	1.03 (±0.34)	0.23 (±0.22)	0.35 (±0.30)
14	0.19 (±0.11)	0.35 (±0.16)	0.12 (±0.12)	0.18 (±0.13)	0.10 (±0.11)	0.18 (±0.09)
Average	0.17 (±0.13)	0.30 (±0.15)	0.43 (±0.22)	0.75 (±0.28)	0.20 (±0.13)	0.49 (±0.33)

The standard deviations of *e*_*PeTra*_ at locations 1, 2, and 3, are 0.13, 0.22, and 0.13 m respectively; which are also slightly lower than *e*_*LD*_ (0.15, 0.28, and 0.33 m respectively).

**Figure 9:**
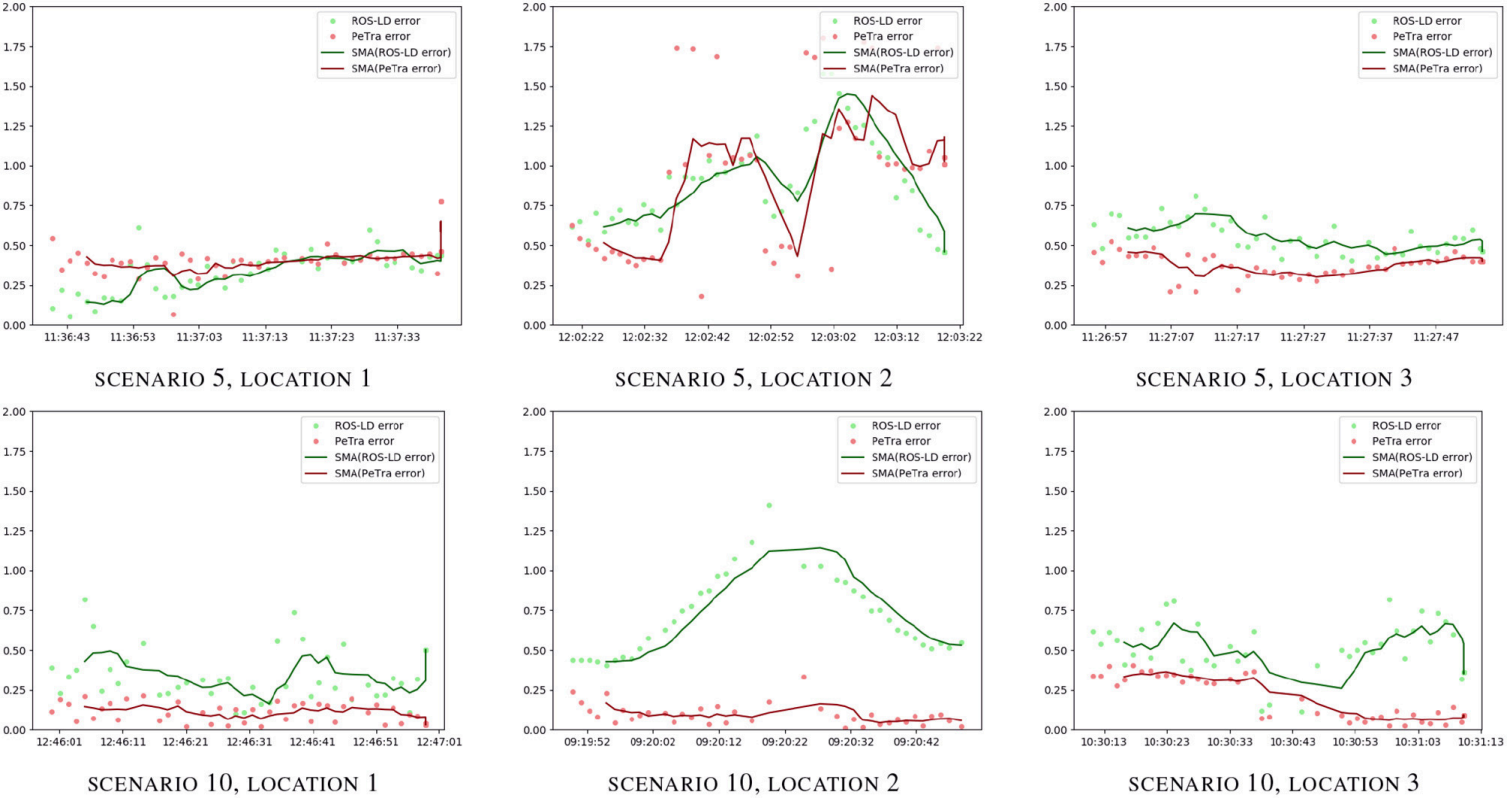
*e*_*PeTra*_ (m) and *e*_*LD*_ (m) evolution over time (hh:mm:ss) on scenarios 5 (1st row), and 10 (2th row), at locations 1 **(left)**, 2 **(center)**, and 3 **(right)**. Red markers show *e*_*PeTra*_, green markers show *e*_*LD*_, red line show (*e*_*PeTra*_), and green line shows MA(*e*_*LD*_).

Figure 9 shows the evolution of the accuracy error for both PeTra and LD over the time horizon given by Rosbag files recorded at locations 1, 2, and 3, on situations 5, and 10, of the second version of the dataset. Green markers represent *e*_*LD*_. Red markers represent *e*_*PeTra*_. Green and red lines illustrate *e*_*LD*_ and *e*_*PeTra*_ Moving Average (MA). MAs were used to smooth out short-term fluctuations and highlight longer-term trends or cycles. The same analysis has been done for all scenarios at all locations. Complete results are shown in Figures S1–S3. According to Table 1, *e*_*PeTra*_ is slightly lower on average than *e*_*LD*_ for scenarios 5 and 10. This is shown in the figure, *e*_*PeTra*_ is lower than *e*_*LD*_ on scenario 5 at location 3, and on scenario 6 at locations 1, 2, and 3. However, there are specific moments where *e*_*LD*_ is lower, especially at scenario 5 on locations 1 and 2.

Table 2 shows the mean error and standard deviation for the two situations of the third version of the dataset. As can be seen in the table, the average error of *e*_*PeTra*_ at the kitchen, the living room, and the bedroom, are 0.18m, 0.13m, and 0.53m, respectively; it is lower than *e*_*LD*_ at the three rooms (0.38, 0.57, and 0.64 m).

**Table 2:** Mean error and standard deviation (m) using data from version 3 of the dataset.

	**Kitchen**		**Living room**		**Bedroom**	
**Situation**	${\bar{X}}_{e_{PeTra}}$	${\bar{X}}_{e_{LD}}$	${\bar{X}}_{e_{PeTra}}$	${\bar{X}}_{e_{LD}}$	${\bar{X}}_{e_{PeTra}}$	${\bar{X}}_{e_{LD}}$
5	0.20 (±0.33)	0.35 (±0.25)	0.14 (±0.17)	0.56 (±0.29)	0.92 (±0.34)	0.98 (±0.33)
10	0.16 (±0.16)	0.40 (±0.19)	0.12 (±0.12)	0.57 (±0.31)	0.13 (±0.12)	0.29 (±0.11)
Average	0.18 (±0.25)	0.38 (±0.22)	0.13 (±0.15)	0.57 (±0.30)	0.53 (±0.23)	0.64 (±0.22)

The standard deviations of *e*_*PeTra*_ at the three rooms are 0.25, 0.15, and 0.23 m respectively are slightly higher than standard deviations of *e*_*LD*_ at the kitchen and at the bedroom (0.22 and 0.22 m) and lower at the living room (0.30 m).

**Figure 10:**
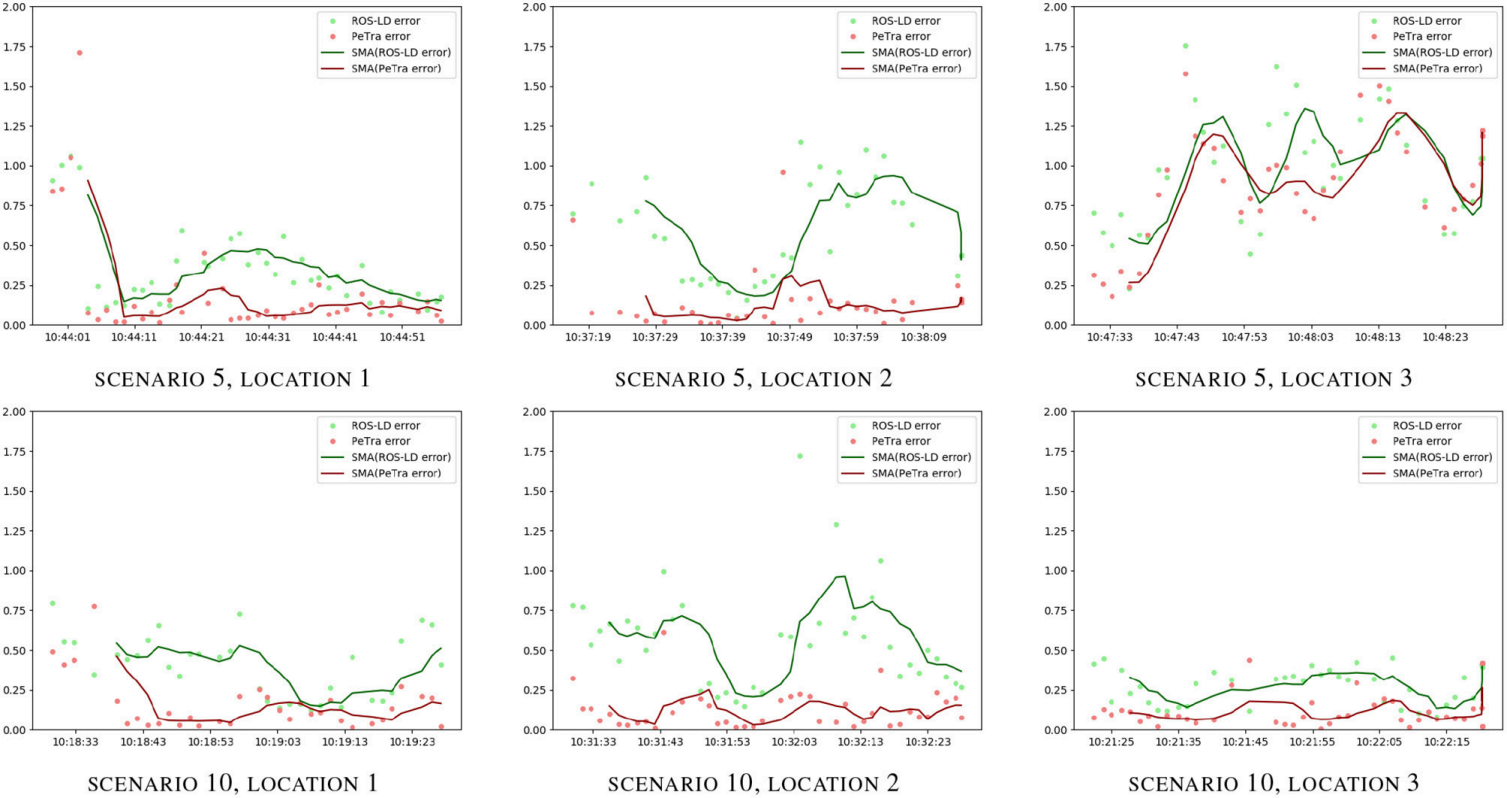
*e*_*PeTra*_ (m) and *e*_*LD*_ (m) evolution over time (hh:mm:ss) on scenarios 5 (1st row), and 10 (2nd row), at locations 1 **(left)**, 2 **(center)**, and 3 **(right)**. Red markers show *e*_*PeTra*_, green markers show *e*_*LD*_, red line shows MA(*e*_*PeTra*_), and green line shows MA(*e*_*LD*_).

Figure 10 shows the evolution of the accuracy error for both PeTra and LD over the time horizon given by rosbag files recorded at locations 1, 2, and 3, on situations 5, and 10, of the third version of the dataset. Green markers represent *e*_*LD*_. Red markers represent *e*_*PeTra*_. Green and red lines illustrate *e*_*LD*_ and *e*_*PeTra*_ MA. According to the figure, *e*_*PeTra*_ is lower than *e*_*LD*_ most of the time for both scenarios.

Regarding performance, PeTra spends ≈0.3 s on calculating a location estimate from LIDAR sensor data when running on Orbi-One hardware configuration as described in 2.1. With the same hardware platform LD spends ≈0.1 s on calculating a location estimate.

## 4. Discussion

Table 1 shows that PeTra has a lower mean error than LD at the three locations. These differences represent an accuracy increase of 43%, 42%, and 59% at locations 1, 2, and 3, respectively. Considering the standard deviation, differences between PeTra and LD represent a reduction of 13, 21, and 60% at locations 1, 2, and 3. Thus, PeTra is more consistent over time at all locations when Orbi-One remains still. Considering each situation, *e*_*PeTra*_ is lower on average than *e*_*LD*_, however, there are not too many differences on multi-person scenarios (scenarios 2, 5, 6 and 9). It is expected because the training data employs a single person.

Figures S1–S3 visually illustrate the evolution over time of *e*_*PeTra*_ and *e*_*LD*_. They show that *e*_*PeTra*_ is lower than *e*_*LD*_ most of the time for most of the scenarios at the three locations analyzed. However, we must analyze carefully the differences between different situations. In order to do so, scenarios 5 and 10 have been selected for in-depth analysis. Graphics of Figure 9 visually illustrate the evolution of *e*_*PeTra*_ and *e*_*LD*_ specifically on these scenarios. These scenarios have been chosen because they include a large variety of situations with one or several people standing or moving around in the environment. They also include situations where PeTra gets a better performance and a worse performance comparing to LD.

**Figure 11:**
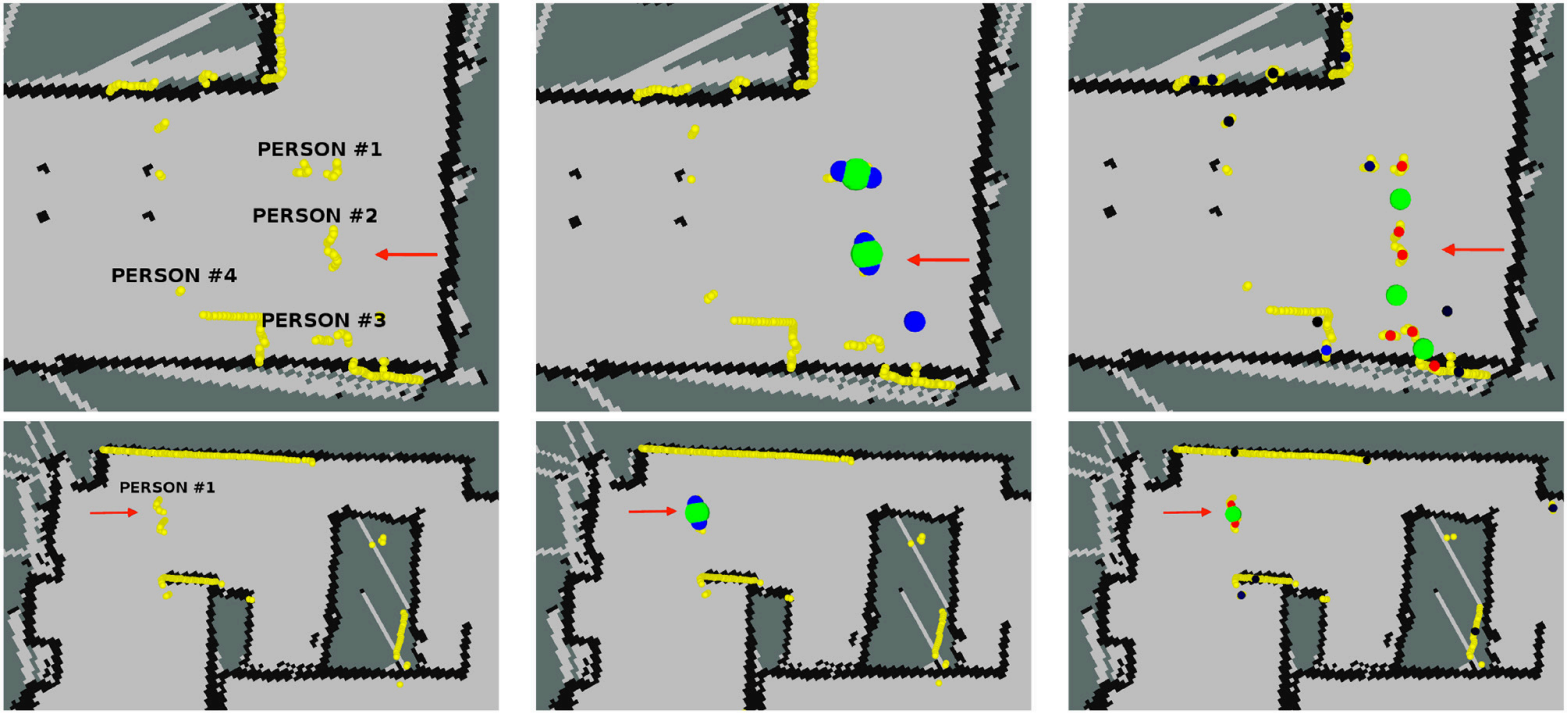
Information of the robot as displayed on Rviz for scenarios 5 (1st row), and 10 (2nd row). Yellow markers show LIDAR readings. The red arrow shows Orbi-One's location and orientation. The beginning of the arrow matches the robot location. PeTra's location estimates are shown on the center: blue markers show leg location estimates and the green marker shows the person's center. LD location estimates are shown on the right: black-to-blue markers show legs location estimates, close-to-black markers represent a low chance it is a real leg, close-to-blue markers represent a high probability; red markers show paired legs; and the green marker shows the person center.

The first row of Figure 11 shows information of the robot on situation 5 at location 2 as displayed at Rviz. On this situation, recorded at the living room of the mock-up apartment, four people stand still in front of the robot while another one moves away. The red arrow shows the robot's pose. The beginning of the arrow matches the robot's location. Yellow markers show LIDAR's readings. These readings allow the identification of the people on the scene. Two half-circles can be guessed in front of the robot at several places in the image. These two half-circles are the shape that the sensor gathers when a person is in the LIDAR range. In this case, PeTra is able to detect two of the people in the scene as shown in Figure 11, top middle. Another person close to the wall is not detected because just one of her legs can be seen. The fourth person is hidden behind another one and is not detected either. Regarding LD, it identifies 3 people in the scene, as shown in Figure 11, top right, but just one of them corresponds to a real person, the others have been located by pairing legs from different people or even from furniture.

The second row of Figure 11 shows information of the robot on situation 10 at location 3. It was recorded at the bedroom of the mock-up apartment. Here, a person first moves away and then moves toward the robot. The LIDAR's readings allow the identification of the person on the scene. In this case, both PeTra and LD identify the person correctly, but LD also identifies some points as people's legs that in fact match furniture legs. These matchings are displayed as black-to-blue markers in Figure 11, bottom right.

Regarding results of Table 2, again *e*_*PeTra*_ has a lower mean error than *e*_*LD*_, representing an accuracy increase of 52, 77, and 18% at locations 1, 2, and 3, respectively. The standard deviation is slightly higher at locations 1 and 3 (12 and 4%) and lower at location 2 (50%). Graphics of Figure 10 visually illustrate the evolution of *e*_*PeTra*_ and *e*_*LD*_ over time, showing that PeTra offers better accuracy than LD most of the time in both situations. These results demonstrate that PeTra is also more consistent over time at all locations when the Robot moves.

## 5. Conclusions and Further Work

This paper presented a system named PeTra to track pair of legs (people) by processing LIDAR sensor data using CNNs. This could be used in several applications, such as improving navigation, facilitating human-robot interaction, or in security and safety. To demonstrate that PeTra allows the tracking of people, it has been compared with LD, a well-known solution for tracking people from LIDAR sensor data, at several locations and situations. Evaluations have been done using data different than the ones used to train and test both models. As a result, experiments proved that PeTra offers a better accuracy in most scenarios.

The main contribution of this work is the PeTra system itself. But in addition, we also want to point out the technical contributions of this work, which include:

1. A people tracking system ready to be used by any mobile robot using the ROS framework.
2. A method to evaluate the performance of range-based people trackers in mobile robots by comparing their results with the data contained in public dataset (RRID:SCR_015743). The dataset is described in section 2.5.1. Data are available at the University of Leon Robotics group website^2^.
3. A system to transform LIDAR sensor data to a two-dimensional occupancy map as described in section 2.3.1, which enables processing them as a picture. This makes LIDAR sensor data treatable by classifiers based on neural networks which used pictures as input.

Regarding further work, there are several aspects that have to be analyzed. On the one hand, a network optimization is needed to improve performance. A mobile robot such as Orbi-One needs to get peoples' locations in real time as soon as LIDAR sensor data is received. A 0.3 s delay can be too muchinon certain situations.

LIDAR sensor data pre-processing could improve performance. Currently, a Boolean matrix is built considering just two situations: in the first one, pointed out as the value 1, LIDAR scan found an obstacle in a given position; in the second one, pointed out as the value 0, LIDAR scan went through it without detecting an obstacle in that position or did not go through it due to an occlusion. Differentiating the cases when the LIDAR scan found an obstacle and when it did not go through that position, could be useful to generate new fit and evaluation data for the CNN used by PeTra.

## Author Contributions

PeTra is based on CÁ-A's work for her End-of-Degree Assignment that was proposed and advised by VM. MC carried out most of the data gathering advised by ÁG-H and CF-L. ÁG-H carried the accuracy evaluation of PeTra. FM and FR-L independently replicated the experiments in their labs. Finally, ÁG-H and VM did the KIO calibration and drafted this manuscript.

### Conflict of Interest Statement

The authors declare that the research was conducted in the absence of any commercial or financial relationships that could be construed as a potential conflict of interest.

## Footnotes

^1^https://www.mrpt.org/robotics_datasets

^2^http://robotica.unileon.es/index.php/Benchmark_dataset_for_evaluation_of_range-based_people_tracker_classifiers_in_mobile_robots

^3^http://robotica.unileon.es/index.php/Testbed

^4^https://www.eu-robotics.net/robotics_league/erl-service/certified-test-beds/index.html

^5^https://youtu.be/Qa6eEJzUlRg

^6^http://www.robotnik.es/manipuladores-roboticos-moviles/rb-one/

^7^http://robotica.unileon.es/~datasets/LegTracking/PeTra_training_dataset/npy_train_test_globales.tar.gz

^8^https://github.com/wg-perception/people

^9^https://www.eu-robotics.net/robotics_league/

^10^http://www.robocup.org/

## Abbreviations

CNN: Convolutional Neural Network
ERL: European Robotics league
INCIBE: Instituto Nacional de Ciberseguridad de España
LD: Leg Detector
LIDAR: Laser Imaging Detection and Ranging
MRPT: Mobile Robot Programming Toolkit
ReLU: Rectified Linear Unit
RNN: Recurrent Neural Network
ROS: Robot Operating System
RTLS: Real Time Location System
MA: Moving Average.
