# Supplementary material for: Tracking People in a Mobile Robot From 2D LIDAR Scans Using Full Convolutional Neural Networks for Security in Cluttered Environments
Source: Front Neurorobot. 2019 Jan 8;12:85. doi: 10.3389/fnbot.2018.00085 (PMC6332292; doi:10.3389/fnbot.2018.00085)
Supplement: Supplementary file 1 [file Data_Sheet_1.PDF]

Supplementary Material: Tracking People in a Mobile Robot from 2D LIDAR scans using Full Convolutional Neural Networks for Security in Cluttered Environments

Ángel Manuel Guerrero-Higueras*, Claudia Álvarez-Aparicio, María Carmen Calvo Olivera, Francisco J. Rodríguez-Lera, Francisco Martín, Vicente Matellán, Camino Fernández-Llamas

*Correspondence:
Ángel Manuel Guerrero-Higueras
am.guerrero@unileon.es

1 SUPPLEMENTARY FIGURES

Figs S1, S2 and, S3 show the evolution of the accuracy error from both systems for each situation over the time horizon given by rosbag files at locations 1, 2, and 3 respectively. Green markers represents e_{LD}. Red markers represents e_{PeTra}. Green and red lines illustrate e_{LD} and e_{PeTra} Moving Average (MA) respectively. They were to smooth out short-term fluctuations and highlight longer-term trends or cycles.

2 VIDEO DEMONSTRATION

In the Youtube channel of the Robotics Group of the University of León readers can find the full video demonstration[1] of PeTra and Leg Detector (LD) working on this scenario displayed on Rviz.

ACRONYMS

MA — Moving Average.

LD — Leg Detector.

[1] https://youtu.be/Qa6eEJzUlRg

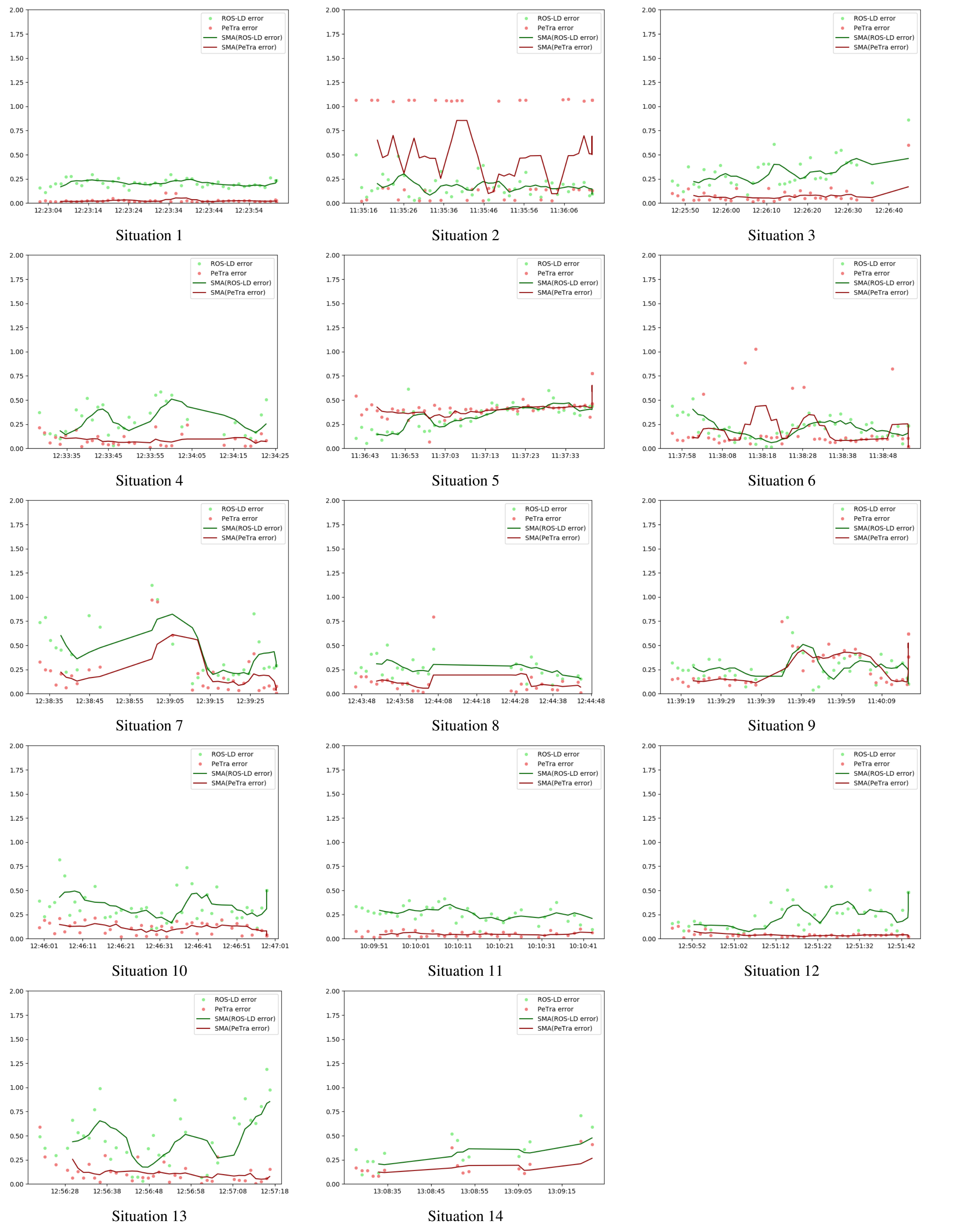

Figure S1. e_{PeTra} (light red line), e_{LD} (light green line), SMA(e_{PeTra}) (dark red line), and SMA(e_{LD}) (dark green line) at location 1.

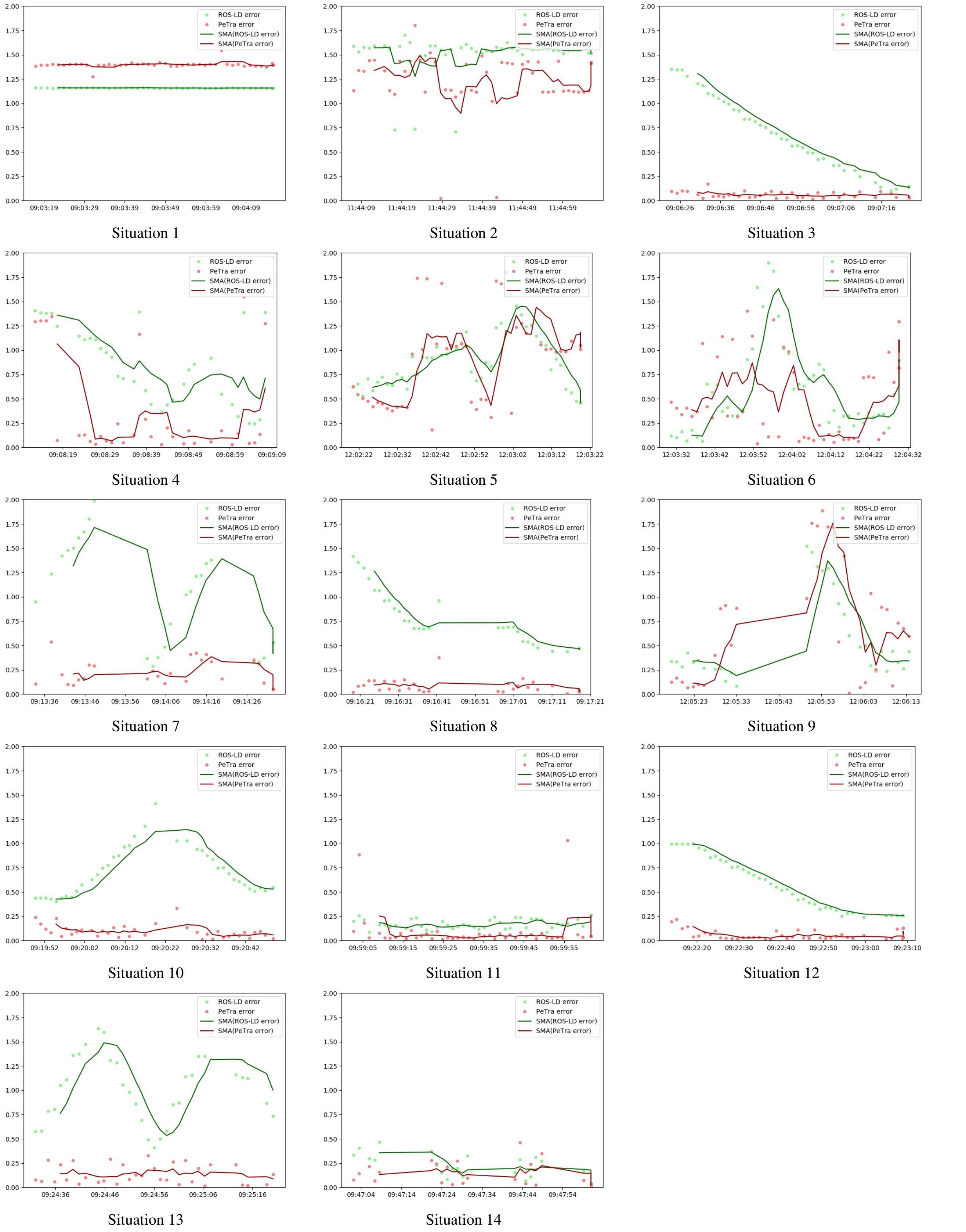

Figure S2. e_{PeTra} (light red line), e_{LD} (light green line), SMA(e_{PeTra}) (dark red line), and SMA(e_{LD}) (dark green line) at location 2.

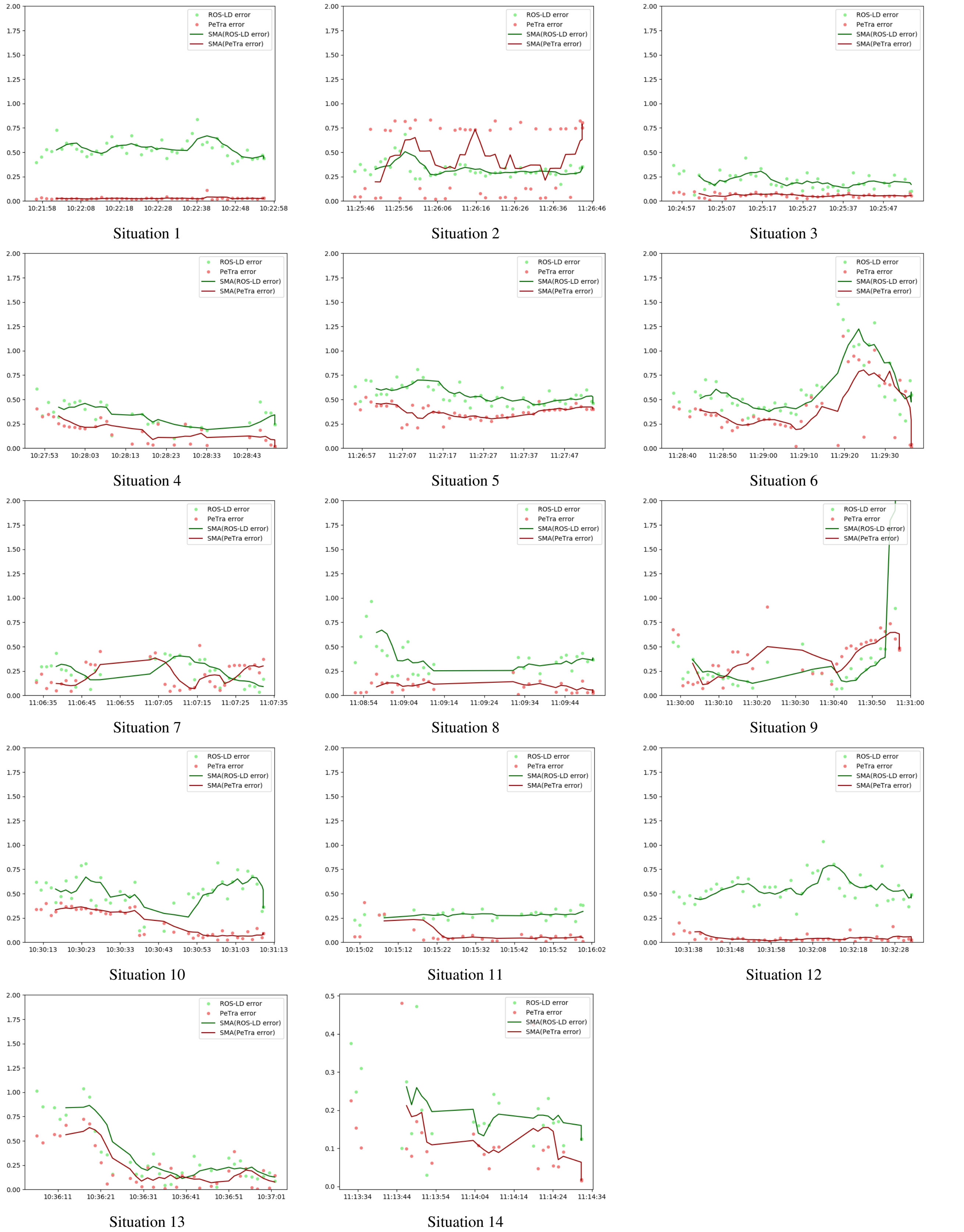

Figure S3. e_{PeTra} (light red line), e_{LD} (light green line), SMA(e_{PeTra}) (dark red line), and SMA(e_{LD}) (dark green line) at location 3.
